# Spontaneous Eye Blink Rate (EBR) Is Uncorrelated with Dopamine D2 Receptor Availability and Unmodulated by Dopamine Agonism in Healthy Adults

**DOI:** 10.1523/ENEURO.0211-17.2017

**Published:** 2017-09-18

**Authors:** Linh C. Dang, Gregory R. Samanez-Larkin, Jaime J. Castrellon, Scott F. Perkins, Ronald L. Cowan, Paul A. Newhouse, David H. Zald

**Affiliations:** 1 Department of Psychology, Vanderbilt University, Nashville, TN 37203; 2 Department of Psychology and Neuroscience, Duke University, Durham, NC 27708; 3 Department of Psychiatry, Vanderbilt University School of Medicine, Nashville, TN 37212; 4 Department of Radiology and Radiological Sciences, Vanderbilt University Medical Center, Nashville, TN 37232; 5 Geriatric Research Education and Clinical Centers, Veterans Administration-Tennessee Valley Healthcare System, Nashville, TN 37212

**Keywords:** dopamine, eye blink rate, PET

## Abstract

Spontaneous eye blink rate (EBR) has been proposed as a noninvasive, inexpensive marker of dopamine functioning. Support for a relation between EBR and dopamine function comes from observations that EBR is altered in populations with dopamine dysfunction and EBR changes under a dopaminergic manipulation. However, the evidence across the literature is inconsistent and incomplete. A direct correlation between EBR and dopamine function has so far been observed only in nonhuman animals. Given significant interest in using EBR as a proxy for dopamine function, this study aimed to verify a direct association in healthy, human adults. Here we measured EBR in healthy human subjects whose dopamine D2 receptor (DRD2) availability was assessed with positron emission tomography (PET)-[18F]fallypride to examine the predictive power of EBR for DRD2 availability. Effects of the dopamine agonist bromocriptine on EBR also were examined to determine the responsiveness of EBR to dopaminergic stimulation and, in light of the hypothesized inverted-U profile of dopamine effects, the role of DRD2 availability in EBR responsivity to bromocriptine. Results from 20 subjects (age 33.6 ± 7.6 years, 9F) showed no relation between EBR and DRD2 availability. EBR also was not responsive to dopaminergic stimulation by bromocriptine, and individual differences in DRD2 availability did not modulate EBR responsivity to bromocriptine. Given that EBR is hypothesized to be particularly sensitive to DRD2 function, these findings suggest caution in using EBR as a proxy for dopamine function in healthy humans.

## Significance Statement

Dopamine is critical for cognitive and reward functions, and dopamine dysfunction is linked to neuropsychiatric disorders including addiction, Parkinson’s disease, and schizophrenia. In humans, direct *in vivo* assessment of the dopamine system is achieved through positron emission tomography (PET). However, PET is costly, labor intensive, exposes participants to radiation, and many research institutes do not have the facilities to conduct human dopamine PET. Spontaneous eye blink rate (EBR) has been proposed as an inexpensive, noninvasive biomarker that can serve as a proxy for dopamine function. Here we present evidence that EBR is not a valid proxy for general dopamine functioning in healthy humans, but it remains to be determined whether EBR can index specific aspects of dopamine functions.

## Introduction

Dopamine is widely studied, with over 5000 publications relating to dopamine function in 2016 alone. Decades of research have revealed the importance of dopamine in cognitive and reward functions, and dopamine dysfunction is linked to disorders including addiction, Parkinson’s disease, and schizophrenia (Ranganath and Jacob, 2016). In humans, direct *in vivo* assessment of the dopamine system is achieved through positron emission tomography (PET; or single photon emission computed tomography). PET together with different radioligands has provided valuable information about different aspects of dopamine function such as receptor density, dopamine release, and dopamine synthesis capacity (Monchi et al., 2006; Buckholtz et al., 2010; Dang et al., 2012). However, each PET scan costs several thousand United States dollars, requires the coordination of multiple specialists (e.g., clinicians and radiochemists), exposes participants to radiation, and many research institutes do not have the radiochemistry or imaging facilities to conduct human dopamine PET. The cost, labor, risk, and opportunity to conduct PET studies have motivated researchers to search for an inexpensive, noninvasive biomarker that can be a proxy for aspects of dopamine function.

One proposed proxy is spontaneous eye blink rate (EBR; Jongkees and Colzato, 2016). Support for an association between dopamine and EBR mainly comes from neuropharmacological studies wherein changes in EBR were observed after administration of dopaminergic agonists or antagonists to animals or human subjects (Elsworth et al., 1991; Lawrence and Redmond, 1991; Kleven and Koek, 1996; Desai et al., 2007; Kaminer et al., 2011). However, as many or more studies reported no effect of dopaminergic manipulation on EBR (Ebert et al., 1996; van der Post et al., 2004; Mohr et al., 2005) or opposite effects of the same dopaminergic drug (Kleven and Koek, 1996; Baker et al., 2002; Kotani et al., 2016), suggesting that the relation between EBR and dopamine might not be as straightforward as some have suggested.

Additional support for the association between EBR and dopamine come from observations of aberrant EBR in individuals with neurologic or psychiatric disorders linked to dopaminergic dysfunction (e.g., Parkinson’s disease and schizophrenia), or a history of using drugs known to affect the dopamine system (e.g., cocaine; Chen et al., 1996; Colzato et al., 2008; Kowal et al., 2011; Fitzpatrick et al., 2012). This evidence is complicated by the fact that aberrant EBR is also present in nondopamine specific conditions such as intellectual disability and traumatic brain injury (Goldberg et al., 1987; Daugherty et al., 1993; Konrad et al., 2003), suggesting that EBR is influenced by and reflective of multiple brain processes (see Jongkees and Colzato, 2016 for a more thorough review of evidence relating EBR to dopamine).

One study has reported a correlation between dopamine D2 receptor (DRD2) and EBR in drug-naive monkeys (Groman et al., 2014). In the study, PET with radioligands for D2 and D1 dopamine receptors were performed on ten vervet monkeys. DRD2 availability positively correlated with baseline EBR and D2-like agonist-induced changes in EBR, suggesting that monkeys with higher DRD2 availability were more sensitive to D2/D3 agonist-induced changes in EBR. Such associations were not observed with D1 receptor availability. These results have not been replicated in humans so it is unclear whether they generalize beyond vervet monkeys. Although nonhuman primates provide a valuable model for studies of the dopamine system, there are notable species differences. Indeed, EBR is almost twice as high in humans compared to vervet monkeys, which could alter its relations with neuropharmacological systems (Tada et al., 2013).

Interest in using EBR as a proxy for dopamine function is substantial, as evidenced by the many studies that use EBR in investigations of associations between dopamine and a range of behavioral responses (Jongkees and Colzato, 2016). However, beyond the varied, and at times contradictory, results regarding the association between EBR and dopamine mentioned above, the majority of evidence for this association, particularly in humans, was observed with neuropharmacological manipulations, neuropsychiatric disorders, and drug use, all of which alter dopamine function such that relations between EBR and dopamine under these conditions may not reflect their association in healthy individuals. The present study used PET with the high affinity DRD2 radioligand [18F]fallypride to examine the predictive power of EBR for DRD2 availability measured *in vivo* in healthy humans. The focus on DRD2 stems from previous results suggesting that EBR is more strongly associated with D2 than D1 receptors (Groman et al., 2014). Additionally, this study examined effects of the dopamine agonist bromocriptine on EBR to determine the responsiveness of EBR to dopaminergic stimulation, and the role of DRD2 in EBR responsivity to bromocriptine.

## Materials and Methods

### Subjects

Twenty healthy subjects between 20 and 50 years old (mean age 33.6 ± 7.6 years, 9F) who had undergone PET-[18F]fallypride for a separate study in our lab were recruited to have their eye blinks recorded for this study, once in a placebo condition and once after bromocriptine administration. Participants were recruited from the Nashville, TN metro area. Exclusion criteria included any history of psychiatric illness on a screening interview (a Structural Interview for Clinical DSM-IV Diagnosis was also available for all subjects and confirmed no history of major Axis I disorders; RRID:SCR_003682; First et al., 1997), any history of head trauma, any significant medical condition, or any condition that would interfere with MRI (e.g., inability to fit in the scanner, claustrophobia, cochlear implant, metal fragments in eyes, cardiac pacemaker, neural stimulator, pregnancy, and metallic body inclusions or other contraindicated metal implanted in the body). Subjects with major medical disorders including diabetes and/or abnormalities on screening comprehensive metabolic panel or complete blood count were excluded. Subjects were also excluded if they reported a history of substance abuse, current tobacco use, alcohol consumption of more than eight ounces of whiskey or equivalent per week, use of psychostimulants (excluding caffeine) more than twice at any time in their life or at all in the past six months, or any psychotropic medication in the last six months other than occasional use of benzodiazepines for sleep. Any illicit drug use in the last two months was grounds for exclusion, even in subjects who did not otherwise meet criteria for substance abuse. Urine drug tests were administered, and subjects testing positive for the presence of amphetamines, cocaine, marijuana, PCP, opiates, benzodiazepines, or barbiturates were excluded. Written informed consent was obtained from all subjects. This study was approved by the Institutional Review Boards at Vanderbilt University and Yale University and performed in accordance with the ethical standards of the 1964 Declaration of Helsinki and its later amendments.

### PET data acquisition

PET imaging was performed on a GE Discovery STE scanner located at Vanderbilt University Medical Center (RRID:SCR_014046). The scanner had an axial resolution of 4 mm and in-plane resolution of 4.5- to 5.5-mm FWHM at the center of the field of view. [18F]fallypride ((S)-N-[(1-allyl-2-pyrrolidinyl)methyl]-5-(3[18F]fluoropropyl)-2,3-dimethoxybenzamide) was produced in the radiochemistry laboratory attached to the PET unit, following synthesis and quality control procedures described in United States Food and Drug Administration IND 47,245. [18F]fallypride is a substituted benzamide with very high affinity to D2/D3 receptors (Mukherjee et al., 1995). 3D emission acquisition scans were performed following a 5.0 mCi slow bolus injection of [18F]fallypride (specific activity >3000 Ci/mmol). CT scans were collected for attenuation correction before each of the three emission scans, which together lasted ∼3.5 h, with two 15-min breaks for subject comfort. PET images were reconstructed with decay correction, attenuation correction, scatter correction, and calibration.

### MRI data acquisition

Structural MRI scans were performed on a 3 Tesla Phillips Achieva scanner located at the Vanderbilt University Institute for Imaging Science. T1-weighted high-resolution 3D anatomic scans (TR = 8.9 ms, TE = 4.6 ms, FOV = 256 × 256, voxel dimensions = 1 × 1 × 1 mm) were obtained for each participant to aid coregistration and spatial normalization of PET images.

### [18F]fallypride binding potential (BP_ND_) image calculation

Voxelwise D2/D3 BP_ND_ images were calculated using the simplified reference tissue model, which has been shown to provide stable estimates of [18F]fallypride BP_ND_ (Siessmeier et al., 2005). The cerebellum served as the reference region because of its relative lack of D2/D3 receptors (Camps et al., 1989). The cerebellar reference region was obtained from an atlas provided by the ANSIR laboratory at Wake Forest University (RRID:SCR_007378). Limited PET spatial resolution introduces blurring and causes signal to spill onto neighboring regions. Because the cerebellum is located proximal to the substantia nigra and colliculus, which both have DRD2, only the posterior 3/4 of the cerebellum was included in the region of interest (ROI) to avoid contamination of [18F]fallypride signal from the midbrain nuclei. The cerebellum ROI also excluded voxels within 5 mm of the overlying cerebral cortex to prevent contamination from cortical signals. The bilateral putamen ROI, drawn according to established guidelines (Mawlawi et al., 2001) on the Montreal Neurological Institute (MNI) brain, served as the receptor-rich region in the analysis. The cerebellum and putamen ROIs were registered to each subject’s T1 image using FMRIB Software Library (FSL) nonlinear registration of the MNI template to each individual subject’s T1. T1 images and their associated cerebellum and putamen ROIs were then coregistered to the mean image of all realigned frames in the PET scan using FSL-FLIRT (RRID:SCR_002823). Emission images from the three PET scans were merged temporally into a 4D file. To correct for motion during scanning and misalignment between the three PET scans, all PET frames were realigned using SPM8 to the frame acquired 10 min after injection (RRID:SCR_007037). Model fitting and BP_ND_ calculation were performed using the PMOD Biomedical Imaging Quantification software (PMOD Technologies). BP_ND_ images represent the ratio of specifically bound ligand ([18F]fallypride in this study) to its free concentration.

**Figure 1.**
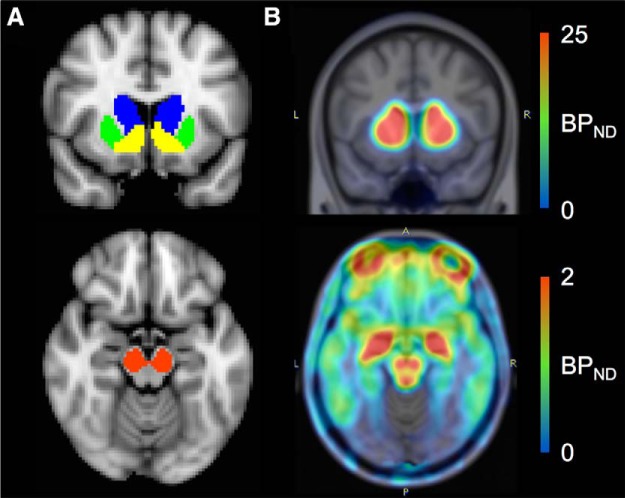
[18F]fallypride BP_ND_ images reflecting DRD2 availability. ***A***, Shown are ROIs from which mean BP_ND_ were extracted for analyses: caudate (blue), putamen (green), ventral striatum (yellow), and midbrain (red). ***B***, Example of a [18F]fallypride BP_ND_ image showing high BP_ND_ in the striatum (top) and midbrain (bottom).

Mean BP_ND_ in the striatum, which has the highest concentration of postsynaptic DRD2 in the brain, and the midbrain, the site of dopamine neurons on which presynaptic DRD2 are located, were extracted and regressed on EBR (Fig. 1). The bilateral midbrain and 3 striatal ROIs (caudate, putamen, and ventral striatum/nucleus accumbens) were drawn in MNI standard space using previously described guidelines (Mawlawi et al., 2001; Dang et al., 2012), registered to PET images using the same transformations for cerebellum registration to PET images, and thresholded at 0.5 after coregistration to exclude voxels on the border that had <50% probability of being part of the ROI, thus ensuring high tissue probability for each ROI masks. Relations between EBR and BP_ND_ outside the striatum and midbrain were examined with an exploratory voxelwise analysis using SPM8 with family wise error correction.

### EBR

Eye blinks were recorded for 5 min using the Pupil Headset (Pupil Labs UG). Five minutes has been proposed as the standard time period for EBR assessment based on tests of reliability and is consistent with the EBR-reading literature from the 1930s and 1940s, where EBR was often reported over a 5-min period (Zaman and Doughty, 1997; Doughty, 2001). Eye blinks were recorded once in the placebo condition and once approximately 4 h after administration of a dopamine agonist, bromocriptine, which is within the time period of maximal bromocriptine effects (Johnson et al., 1976; Di Chiara et al., 1978; Pizzolato et al., 1985). Bromocriptine was administered at a dose of 1.25 mg, a typical amount used in studies of bromocriptine effects on humans (Mehta et al., 2001; Cools et al., 2007; McAllister et al., 2011). Subjects were instructed to sit back, relax, and look forward but were not instructed to focus on a particular point to minimize active control of eye movements. During the recording of eye blinks, subjects were in a quiet room with one other person (the experimenter). In accordance with protocols for protecting human subjects, an experimenter was present with the subject at all times during the study session to monitor possible negative side effects from bromocriptine. Subjects were aware that their eye blinks were recorded as they had to wear the eye tracking device like a pair of glasses. Subjects were told that eye blinks were recorded to examine the relation between spontaneous EBR and dopamine function but did not receive any instruction regarding blinking. Subjects were given as much time as they needed (typically 1–3 min) after putting on the eye tracking device to become comfortable wearing the device, but the protocol did not include a habituation period. EBR recordings were performed around noon if the study session started in the morning, and around 5 P.M. if the study session started after noon. Although there is minimal diurnal variation in spontaneous EBR from early to late afternoon (Barbato et al., 2000), the start times were kept consistent across sessions (i.e., each subject started both study sessions in the morning or both in the afternoon).

Subjects were asked to remove contact lenses before the recording of eye blinks if they wore contact lenses. Placebo/bromocriptine session order, blind to both the subject and the researcher, was counterbalanced across subjects. Eye blinks were visually counted with interrater and intrarater reliability above 95%. EBR was defined as the number of eye blinks per minute. EBR data from the bromocriptine condition were not available for two subjects: data from one subject were lost due to a technical failure, and data from another subject were excluded from analysis because the subject reported eye irritation after removing contact lenses and blinked excessively during the recording of eye blinks. Eye blink recording for one subject in the bromocriptine condition inadvertently terminated at 4 min, and thus EBR was calculated using 4 min of data for this session.

An average of 17 months (range: 3–32 months) separated the PET-[18F]fallypride scan from the recording of eye blinks. The time lag reflected that the majority of subjects were recruited for the EBR and bromocriptine study after having already completed the PET study, and the expense of PET data collection did not allow collection of a new cohort of participants. Time difference in data acquisition along with age and sex were entered as covariates in all regressions of [18F]fallypride BP_ND_ on EBR; standardized beta coefficients (correlations), *t* statistics, and *p* values for the relations between [18F]fallypride BP_ND_ and EBR from these regressions are reported in Results.

**Figure 2.**
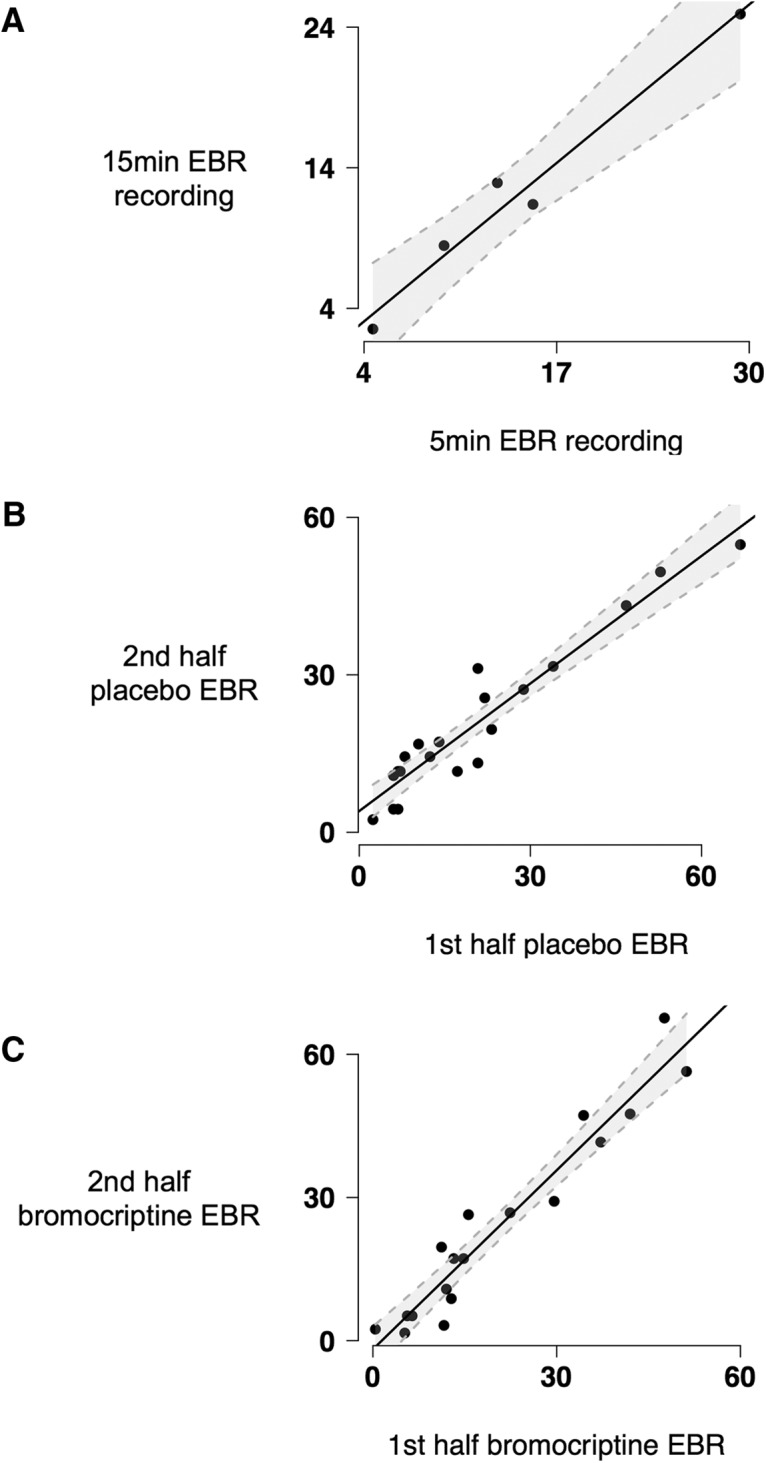
Lengths of EBR recording. ***A***, EBR in the first 5 min of recording strongly correlated with EBR over the entire 15 min of recording (*r*_3_ = 0.98, *p* = 0.002). ***B***, ***C***, EBR from the first and latter half of each subject’s 5-min EBR recording also correlated very strongly in both the placebo (*r*_18_ = 0.96, *p* = 4.9 × 10^−11^) and bromocriptine (*r*_16_ = 0.84, *p* = 1.2 × 10^−5^) conditions.

Five-minute recordings of spontaneous EBR are generally viewed as providing a representative sample of behavior, as even shorter measurement windows have been shown to be stable when assessed repeatedly over the course of an hour-long session (Brezinová and Kendell, 1977) if subjects were not visually engaged with a narrative or intervening tasks or distractions (Nakano et al., 2009). The 5-min duration of EBR recording in this study was similar, and even longer, than the time windows used by previous studies assessing effects of dopamine on EBR (Semlitsch et al., 1993; Cavanagh et al., 2014). Nonetheless we confirmed that EBR can be assessed reliably in 5 min using two different approaches. In the first approach, to confirm that EBR in an initial 5-min window was representative of EBR over a longer period (e.g., 15 min), we recruited five healthy subjects to undergo eye blink recording for 15 min. These subjects received the same instructions for eye blink recording as subjects in the bromocriptine/placebo study. EBR in the first 5 min of recording strongly correlated with EBR over the entire 15 min of recording (*r*_3_ = 0.98, *p* = 0.002)^a^, providing evidence that 5 min was sufficient to capture spontaneous EBRs reliably. In the second approach, we separately calculated EBR for the first and latter half of each subject’s placebo and bromocriptine session’s 5-min EBR recording. The two EBR measures correlated very strongly in both the placebo (*r*_18_ = 0.96, *p* = 4.9 × 10^−11^)^b^ and bromocriptine (*r*_16_ = 0.84, *p* = 1.2 × 10^−5^)^c^ conditions. Results in this study observed using EBR calculated over 5 min still held when EBR was calculated in half that time window, showing that EBR was very stable and can even be assessed in under 5 min (Fig. 2), Table 1.

**Table 1.** Statistical table

Line	Data/dependent variable^*^	Type of test	Statistic	Confidence
a	15 min EBR ∼ 5 min EBR	Pearson's correlation	*r* = 0.98, dof =3	*p* = 0.002
b	Placebo: 1st half EBR ∼ 2nd half EBR	Pearson's correlation	*r* = 0.96, dof =18	*p* < 0.0001
c	Bromocriptine: 1st half EBR ∼ 2nd half EBR	Pearson's correlation	*r* = 0.84, dof =16	*p* < 0.0001
d	Placebo EBR	Dixon's test	*Q* = 0.30	*p* = 0.597
e	Bromocriptine EBR	Dixon's test	*Q* = 0.22	*p* = 0.908
f	Baseline EBR ∼ caudate BP_ND_	Linear regression	*t* = −0.67, dof = 15	*p* = 0.512
g	Baseline EBR ∼ putamen BP_ND_	Linear regression	*t* = −0.76, dof = 15	*p* = 0.461
h	Baseline EBR ∼ ventral striatum BP_ND_	Linear regression	*t* = 0.95, dof = 15	*p* = 0.356
i	Baseline EBR ∼ midbrain BP_ND_	Linear regression	*t* = 0.14, dof = 15	*p* = 0.890
j	Baseline EBR ∼ whole brain BP_ND_	Linear regression	No significant cluster	*p* = 0.05 corrected for FWE
k	Baseline EBR, bromocriptine EBR	Pearson's correlation	*r* = 0.83, dof = 16	*p* < 0.0001
l	Baseline EBR, bromocriptine EBR	Paired *t* test	*t* = 0.35, dof = 17	*p* = 0.734
m	Changes in EBR ∼ body weight	Linear regression	*t* = −0.16, dof = 13	*p* = 0.877
n	Changes in EBR ∼ caudate BP_ND_	Linear regression	*t* = −1.50, dof = 13	*p* = 0.157
o	Changes in EBR ∼ putamen BP_ND_	Linear regression	*t* = −1.35, dof = 13	*p* = 0.199
p	Changes in EBR ∼ midbrain BP_ND_	Linear regression	*t* = −0.11, dof = 13	*p* = 0.912
q	Changes in EBR ∼ ventral striatum BP_ND_	Linear regression	*t* = −2.06, dof = 13	*p* = 0.060
r	Changes in EBR ∼ caudate BP_ND_	Quadratic regression	*t* = −0.06, dof = 12	*p* = 0.951
s	Changes in EBR ∼ putamen BP_ND_	Quadratic regression	*t* = 1.88, dof = 12	*p* = 0.085
t	Changes in EBR ∼ ventral striatum BP_ND_	Quadratic regression	*t* = 1.18, dof = 12	*p* = 0.260
u	Changes in EBR ∼ midbrain BP_ND_	Quadratic regression	*t* = 0.15, dof = 12	*p* = 0.882

* age, sex, and time difference were covariates in all multiple regressions.

## Results

As expected, there were significant individual differences in spontaneous EBR (mean 21 ± 16 on placebo, and mean 23 ± 18 on bromocriptine). The Dixon’s test for outliers confirmed that there were no outliers in the placebo condition (*Q* = 0.30, *p* = 0.597)^d^ and the bromocriptine condition (*Q* = 0.22, *p* = 0.908)^e^. All subjects were therefore included in primary analyses. To correct for multiple comparisons of four ROIs, results were considered significant at *p* < 0.0125.

### Baseline EBR and DRD2 availability

**Figure 3.**
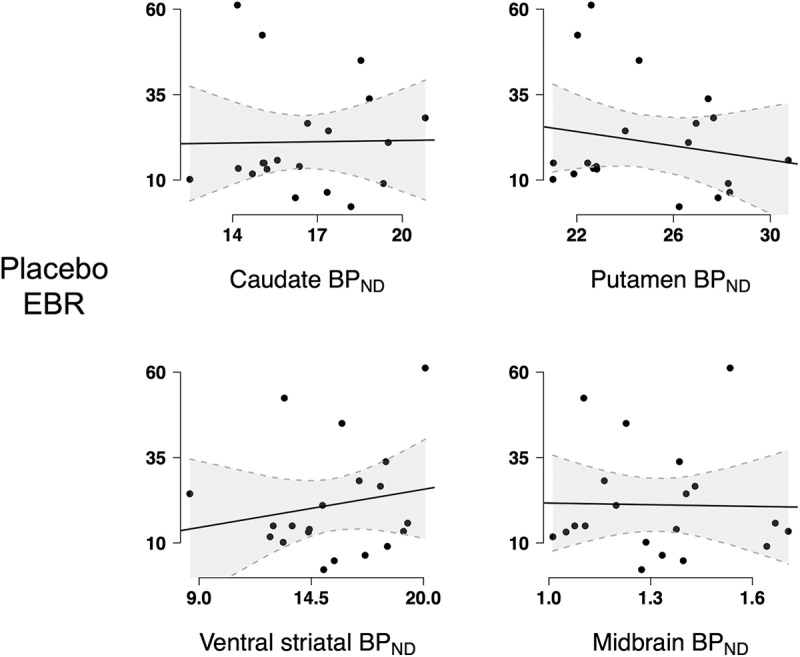
EBR and [18F]fallypride BP_ND_. EBR in the placebo condition did not significantly correlate with [18F]fallypride BP_ND_ in the caudate (*t*_15_ = –0.67, *p* = 0.512), putamen (*t*_15_ = –0.76, *p* = 0.461), ventral striatum (*t*_15_ = 0.95, *p* = 0.356), or midbrain (*t*_15_ = 0.14, *p* = 0.890).

EBR in the placebo condition did not significantly relate to [18F]fallypride BP_ND_ in the caudate (β = –0.21, *t*_15_ = –0.67, *p* = 0.512)^f^, putamen (β = –0.22, *t*_15_ = –0.76, *p* = 0.461)^g^, ventral striatum (β = 0.24, *t*_15_ = 0.95, *p* = 0.356)^h^, or midbrain (β = 0.04, *t*_15_ = 0.14, *p* = 0.890)^i^ (Fig. 3), Table 1. Voxelwise analysis did not identify any significant association between EBR and BP_ND_ outside the striatum and midbrain, in addition to confirming the lack of such association in the striatum and midbrain^j^.

### Effects of bromocriptine on EBR

**Figure 4.**
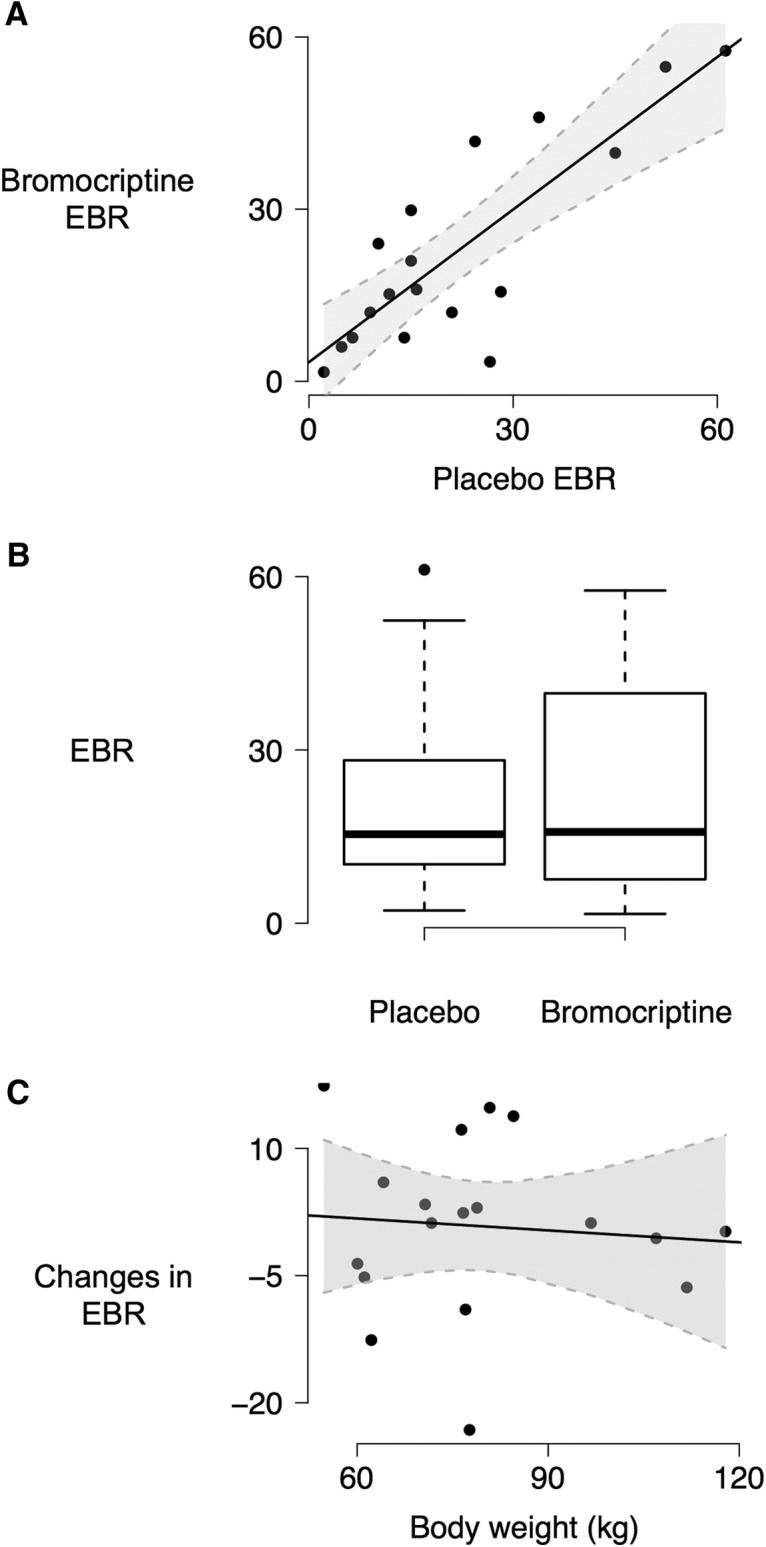
Bromocriptine and EBR. EBR in the placebo and bromocriptine conditions were highly correlated (*r*_16_ = 0.83, *p* < 0.0001; ***A***) but did not differ significantly (*t*_17_ = 0.35, *p* = 0.734; ***B***). ***C***, Body weight did not correlate with bromocriptine-induced changes in EBR (*t* = –0.16, *p* = 0.878).

EBR in the bromocriptine condition was highly correlated with EBR in the placebo condition (*r*_16_ = 0.83, *p* < 0.0001)^k^ (Fig. 4*A*), indicating reasonable test-retest reliability despite the drug challenge. However, EBR in the placebo condition did not differ significantly from EBR in the bromocriptine condition (*t*_17_ = 0.35, *p* = 0.734, 95% CI [–10.9, 12.6])^l^ (Fig. 4*B*). Because we used a fixed dose of bromocriptine, there may be a negative relationship between body weight and the resulting blood plasma levels and CNS actions of bromocriptine. However, there was no association between body weight and bromocriptine-induced changes in EBR (β = –0.06, *t* = –0.16, *p* = 0.877)^m^ in the present data (Fig. 4*C*).

Groman et al. (2014) observed that monkeys with high DRD2 availability exhibited greater D2-like (D3 preferring PHNO) drug-induced increases in EBR, with those low in DRD2 availability even showing declines in EBR. To examine whether DRD2 availability positively related to bromocriptine-induced changes in EBR, we regressed [18F]fallypride BP_ND_ on the difference in EBR between the placebo and bromocriptine conditions. Bromocriptine effects on EBR were not significantly predicted by BP_ND_ in the caudate (β = –0.52, *t*_13_ = –1.50, *p* = 0.157)^n^, putamen (β = –0.48, *t*_13_ = –1.35, *p* = 0.199)^o^, or midbrain (β = –0.03, *t*_13_ = –0.11, *p* = 0.912)^p^. Ventral striatal BP_ND_ had the largest association with bromocriptine-induced changes in EBR out of the four ROIs but was not statistically significance even at the uncorrected level (β = –0.52, *t*_13_ = –2.06, *p* = 0.060)^q^, Table 1. While this ventral striatal result might be considered equivocal in a study with modest statistical power, it is critical to note that the observed relationship was in the opposite direction than predicted, with EBR decreasing in individuals with the highest ventral striatal BP_ND._ Bromocriptine effects on EBR also did not relate to BP_ND_ in any ROI when changes in EBR were calculated as the percentage change from EBR in the placebo condition (all *p* > 0.10).

The influence of dopamine on behavior has been proposed to have an inverted-U profile in which individual differences in baseline dopamine function nonlinearly affect individual responses to dopaminergic stimulation. To examine this hypothesis in our data, we performed quadratic regressions of [18F]fallypride BP_ND_ on bromocriptine-induced changes in EBR. There was no significant parabolic relation between [18F]fallypride BP_ND_ and changes in EBR: caudate (*t*_12_ = –0.06, *p* = 0.951)^r^, putamen (*t*_12_ = 1.88, *p* = 0.085)^s^, ventral striatum (*t*_12_ = 1.18, *p* = 0.260)^t^, or midbrain (*t*_12_ = 0.15, *p* = 0.882)^u^, Table 1.

## Discussion

The present results showed no relation between EBR and DRD2 availability in healthy human subjects. EBR also was not responsive to mild dopaminergic stimulation by bromocriptine in a consistent manner across subjects, and individual differences in DRD2 availability did not substantially modulate EBR responsivity to bromocriptine. Given that EBR is hypothesized to be particularly sensitive to DRD2 (Groman et al., 2014), these findings suggest caution in using EBR as a proxy for dopamine function in healthy humans.

Most studies that have reported a relation between EBR and dopamine function observed the association in atypical populations (e.g., individuals with psychiatric or neurologic conditions or a history of drug use) or under a neuropharmacological manipulation (Jongkees and Colzato, 2016). EBR and dopaminergic function may be correlated in clinical conditions at the “extremes” of dopaminergic functioning wherein the linkage becomes evident when the dopamine system is significantly damaged or dysregulated. Our data suggest that the influence of dopamine (specifically DRD2) on EBR is limited within healthy humans. The dopamine system comprises multiple feedback loops that, in response to deviation from regular dopamine functioning, could alter relations between different aspects of the dopamine system and their associations with behavior (Cooper et al., 2003). For example, in older adults, compensatory changes in dopamine function alter the relation between dopamine function and brain activation during task performance and cognitive outcomes (Braskie et al., 2008; Braskie et al., 2011).

It is worth noting that several studies employing neuropharmacological approaches have reported no effects of dopaminergic drugs on EBR (Ebert et al., 1996; van der Post et al., 2004; Mohr et al., 2005). Also arguing against the use of EBR as an index of general dopamine functioning are data showing that not all agonists increase EBR and not all antagonists decrease EBR (Jongkees and Colzato, 2016). Consistent with other studies (Depue et al., 1994; Ebert et al., 1996), the present study did not observe an overall effect of bromocriptine on EBR. Interestingly among human studies with D2 agonists, the only study to observe effects was a study by Cavanagh et al. (2014). Using the agonist Cabergoline, this effect only emerged when they split the subjects into high and low blinkers with the low blinkers showing increases and the high blinkers showing decreases. We did not observe a similar inverted-U profile of individual differences in DRD2 availability affecting EBR responses to bromocriptine. It should be noted that in the present study, we administered a low dose of bromocriptine (1.25 mg) to minimize gastrointestinal side effects, which may have limited the impact of bromocriptine on EBR. A complication of low doses of D2 agonists is that they may stimulate autoreceptors that act to lower endogenous dopamine release rather than causing a simple stimulation of postsynaptic D2 receptors (Grace, 1995). However, previous studies administering higher doses of bromocriptine (2.5 mg) also observed no overall effect of bromocriptine on EBR (Depue et al., 1994; Ebert et al., 1996). A separate study showed that a levodopa equivalent dose 20 times higher than the dose in this study and more than twice the dose administered by Cavanagh and colleagues still had no effect on EBR (Mohr et al., 2005). EBR may relate to certain aspects of dopamine function rather than reflective of general dopamine functioning. Given that different components of the dopamine system are differentially associated with pathology and behavior (Cools et al., 2006; Dang et al., 2017), an understanding of the specificity of dopamine effects on EBR would enhance the usefulness of EBR as a proxy for dopamine function.

The primary limitation of this study is the small sample size, although the current sample size is comparable to typical PET studies and larger than most studies assessing the relation between EBR and dopamine (Jongkees and Colzato, 2016). However, for EBR to be a reliable proxy for, and predictor of, dopamine function, the correlation between EBR and dopamine function should be quite large and detectable at the current sample size. Another limitation is that PET-[18]fallypride data were acquired months before eye blink data. Although this time difference was controlled for in all analyses involving [18]fallypride BP_ND_ and EBR, we cannot dismiss the possibility that there may have been changes in dopamine function during this time that altered the relation between DRD2 availability and EBR in a manner not accounted for by the time difference. Published data on the long-term stability of [18F]fallypride binding is lacking at present. However, individual differences in D2-like receptor availability as measured by [18F]fallypride are stable across time periods of a month or more and thus appears to provide a reasonably stable index of individual differences in striatal dopamine D2-like function (Mukherjee et al., 2002).

Regarding the assessment of EBR, we note that Groman et al. (2014) recorded eye blinks for 60 min in their study of drug-naive monkeys, whereas we used a far briefer 5-min measurement. Previous studies assessing effects of dopaminergic drugs on EBR have used similar or shorter time windows as used here (Semlitsch et al., 1993; Cavanagh et al., 2014). Such brief EBR assessment has been shown to have high test-retest reliability (Kruis et al., 2016). In the present work, EBR both within (split-half), and across the placebo and bromocriptine conditions were highly correlated, which shows that EBR can be reliably assessed in 5 min. Moreover, in an independent sample, EBR in the first 5 min of recording also strongly correlated with EBR assessed over 15 min, providing evidence that EBR measured over 5 min is representative of EBR over a longer time period. It may be that, in individuals with intact dopamine functioning, the relationship between EBR and DRD2 availability is subtle and requires far longer assessment of EBR to materialize. However, if the relation between EBR and DRD2 availability were subtle enough that even modest confounds or measurement error obfuscate it, there should be caution in using EBR as a simple, quick proxy for dopamine function.

We note that although [18F]fallypride BP_ND_ is generally interpreted as representing DRD2 availability (especially given the high affinity of [18F]fallypride for DRD2), [18F]fallypride BP_ND_ is also influenced by endogenous dopamine levels (with higher dopamine causing lower BP_ND_ because [18F]fallypride competes with endogenous dopamine for DRD2). The observation of low EBR in Parkinson’s disease patients suggests that EBR might correlate with tonic dopamine levels, which are more closely indexed by PET tracers for dopamine synthesis rather than dopamine receptor availability. Future studies assessing the relation between EBR and dopamine synthesis might clarify this possibility. We additionally note that [18F]fallypride binds to both D2 and D3 receptors and weakly to D4 receptors. If EBR is specifically mediated by a particular type of dopamine receptor, the nonspecificity of [18F]fallypride within the D2 family of receptors might obscure the relationship between EBR and [18F]fallypride BP_ND_. However, it should be noted that we did not observe different patterns of association across striatal regions despite their differing levels of relative D2 and D3 expression.

In conclusion, the present findings suggest that EBR is not a valid proxy for general dopamine functioning in healthy humans, but it remains to be determined whether EBR can index specific aspects of dopamine functions.

## Synthesis

Reviewing Editor: Philippe Tobler, University of Zurich Decisions are customarily a result of the Reviewing Editor and the peer reviewers coming together and discussing their recommendations until a consensus is reached. When revisions are invited, a fact-based synthesis statement explaining their decision and outlining what is needed to prepare a revision will be listed below. The following reviewer(s) agreed to reveal their identity: Heleen Slagter, Philippe Tobler

The authors have adequately addressed the previously raised issues, with one exception.

Major point: One cannot place a lot of confidence into the early-late correlation because it is based on just 5 subjects (even though the correlation is close to 1). Given how quickly the authors collected the 15-min of sEBR in these 5 subjects, why not increase the sample for this analysis to e.g., n=40 so that more confidence can be placed in this critical result? If the result replicates in this larger sample this could also make this paper THE reference for future papers to justify length of EBR collection. Although the split-half results indicate the 5-min recording is reliable, these don't address the question as to whether longer recordings may better capture dopaminergic activity, given the previously shown changes in blink rate over time. If further data collection is not feasible, in the minimum it should be explicitly stated that the early-late correlation will have to be confirmed with a much larger sample.

Minor points:

- The authors should indicate whether the additionally recorded subjects were given the same instructions as the original ones.

- The font types used in figures should be unified.

## Author Response

The authors have adequately addressed the previously raised issues, with one exception.

Major point: One cannot place a lot of confidence into the early-late correlation because it is based on just 5 subjects (even though the correlation is close to 1). Given how quickly the authors collected the 15-min of sEBR in these 5 subjects, why not increase the sample for this analysis to e.g., n=40 so that more confidence can be placed in this critical result? If the result replicates in this larger sample this could also make this paper THE reference for future papers to justify length of EBR collection. Although the split-half results indicate the 5-min recording is reliable, these don't address the question as to whether longer recordings may better capture dopaminergic activity, given the previously shown changes in blink rate over time. If further data collection is not feasible, in the minimum it should be explicitly stated that the early-late correlation will have to be confirmed with a much larger sample.

These comments suggested to us that we did not provide enough scientific background for the assessment and stability of EBR in our manuscript. After consulting with the editor (Prof. Philippe Tobler), we revised the manuscript to reference the following studies in the Methods to contextualize our approach within the recommended method for EBR assessment (lines 244-248 and 293-298).

5 minute has been proposed as the standard time period for EBR assessment based on tests of reliability and is consistent with the EBR-reading literature from the 1930s and 1940s, where EBR was often reported over a 5-min period (Zaman and Doughty, 1997; Doughty, 2001). Regarding the stability of EBR over longer recordings, eye blink pattern has been noted to remain highly stable if recording conditions were kept constant since the seminal paper on eye blinks (Ponder and Kennedy, 1928). We note that Ponder and Kennedy measured blinks by attaching threads to participants' eyelids; this intrusive apparatus required 5-10 minutes of adjustment and thus an initial adaptation period was necessary for their study, but there is little experimental evidence to support a case for an adaptation period with nonintrusive methods such as video recording (Zaman and Doughty, 1997; Doughty, 2001). EBR has been shown to be stable for up to an hour when subjects passively viewed visual scenes devoid of any narrative (Brezinova and Kendell, 1977). In fact, a study with a design similar to our examination of EBR in 5min vs 15min has been previously conducted. In the study (Nakano et al., 2009), eye blinks were recorded during 3.5min of rest followed by three 3.5min-periods of viewing scenes or listening to an audio clip; the periods of viewing scenes or listening to an audio clip were separated by 1min of rest. Across 16min of each experimental condition, EBR did not differ between the initial rest and when subjects passively viewed videos of landscapes or tropical fishes, or listened to an audio book. EBR in the study only changed when subjects viewed videos with plot development, consistent with findings that EBR is highly stable unless subjects are visually engaged with a narrative or intervening tasks or distractions (e.g. reading or performing visual search), with task difficulty linked to the magnitude of EBR change (Stern et al., 1984).

Minor points:

- The authors should indicate whether the additionally recorded subjects were given the same instructions as the original ones.

Yes, the additionally recorded subjects received the same instructions as the original subjects. This information is now in the Methods (lines 305-307).

- The font types used in figures should be unified.

We have revised the fonts as suggested.

References

Brezinova V, Kendell RE (1977) Smooth pursuit eye movements of schizophrenics and normal people under stress. Br J Psychiatry 130:59-63.

Doughty MJ (2001) Consideration of three types of spontaneous eyeblink activity in normal humans: during reading and video display terminal use, in primary gaze, and while in conversation. Optometry Vision Sci 78:712-725.

Nakano T, Yamamoto Y, Kitajo K, Takahashi T, Kitazawa S (2009) Synchronization of spontaneous eyeblinks while viewing video stories. P R Soc B 276:3635-3644.

Ponder E, Kennedy WP (1928) On the act of blinking. Q J Exp Physiol 18:88-109.

Stern JA, Walrath LC, Goldstein R (1984) The endogenous eyeblink. Psychophysiology 21:22-33.

Zaman ML, Doughty MJ (1997) Some methodological issues in the assessment of the spontaneous eyeblink frequency in man. Ophthal Physl Opt 17:421-432.
